# Benefits of automated pig feeding system: A simplified cost–benefit analysis in the context of Kazakhstan

**DOI:** 10.14202/vetworld.2023.2205-2209

**Published:** 2023-11-01

**Authors:** Gulmira K. Dambaulova, Vladimir A. Madin, Zheniskul A. Utebayeva, Madina K. Baimyrzaeva, Leila Z. Shora

**Affiliations:** 1 Regional Smart-Center, A. Baitursynov Kostanay Regional University, Kostanay, Kazakhstan; 2 Department of Software Development and Maintenance, A. Baitursynov Kostanay Regional University, Kostanay, Kazakhstan; 3 Department of Accounting and Management, A. Baitursynov Kostanay Regional University, Kostanay, Kazakhstan; 4 Department of Economic and General Education Disciplines, Eurasian Law Academy named after D.A. Kunaev, Almaty, Kazakhstan

**Keywords:** automated feeding system, cost–benefit analysis, efficiency, pig farming

## Abstract

**Background and Aim:**

Automated pig feeding system is an emerging technology with the potential to considerably enhance pig farming. This study aimed to explore the benefits of automated pig feeding systems and provide a simplified cost–benefit analysis, which would serve as a valuable decision-making tool for the stakeholders.

**Materials and Methods:**

This study conducted a literature review of automated pig feeding systems and explored recent advancements. We conducted a cost–benefit analysis to assess the economic feasibility of implementing an automated feeding system in pig farming. Finally, the case study site, a pig farm in Kazakhstan, has been introduced to provide key information.

**Results:**

The results described an automated pig feeding system suitable for a farm with 500 pigs in Kazakhstan. The case study was further enhanced using a simplified cost–benefit analysis tailored to the farm’s needs and circumstances.

**Conclusion:**

The designed automated pig feeding system is a marked advancement that seamlessly integrates the currently available automation and management technologies. Its distinguishing feature is the inclusion of remote control capabilities and real-time data provision, which utilize modern technology to transform pig farming management.

## Introduction

Over the past five decades, there has been a global shift in the preference for primary sources of food, moving away from grains and toward animal proteins, which has led to a considerable increase in livestock production worldwide [1]. With the increase in demand for pork, the need for innovative and efficient animal feeding systems that can optimize productivity, enhance animal welfare, and improve overall farm profitability has also increased. In recent years, the advancement of technologies such as PlayStation, Xbox, and similar platforms has provided an impetus for the sophistication of automated pig feeding systems. These systems utilize precise feeding techniques to deliver individualized and precise amounts of feed tailored to the specific requirements of each pig. This approach ensures optimal nutrition and improves the feeding outcomes [2]. At the same time, the current technology is limited in its decision support tools and has the capability to feed pig groups based solely on their weights [3]. Drawing from available technological advances, Slader and Gregory [4] conducted a study in which they designed and tested an automated pig feeding system. This study aimed to establish feeding patterns in group-fed pigs. The success of their study generated considerable interest from research agencies and commercial companies, fueling the further development and exploration of automated pig feeding systems. In Kazakhstan, the pig breeding industry is considered non-traditional, with a relatively small domestic pig sector consisting of <1,000,000 pigs and an average density of 0.34 pigs/km^2^ [5]. However, despite its size, there is a potential for expanding the export of pork products [6], driven by the growing demand in the importing markets because of restrictions on countries affected by African swine fever [5]. In the economic development of pig farming, the availability of high-quality feed that is efficiently utilized plays a crucial role because feed costs typically account for a considerable portion of the cost structure of livestock products, reaching approximately 65%–70% [7]. Dambaulova *et al*. [8] reported that one of the main factors contributing to the natural loss of pigs is the irrational organization of animal feeding, which may include inadequate nutrition, poor feed quality, improper feeding schedules, or inconsistent feeding practices. Notably, one of the fundamental principles of animal welfare is “good feeding,” which refers to providing animals with appropriate and high-quality nutrition to promote comfort and overall well-being [9].

This study addresses a major obstacle faced by farms in Kazakhstan. Despite the country’s abundant grain production and potential fodder supply, its agricultural industry is hindered by deteriorating infrastructure and outdated material resources. This challenge is exacerbated by the absence of scientifically validated and practically tested technologies [10]. Therefore, there is an urgent need to develop suitable, practically applicable technologies.

This study aimed to propose the implementation of an automated pig feeding system on a farm in Kazakhstan and evaluate its efficiency through simplified calculations of a cost–benefit analysis. This study considers the specific characteristics and requirements of the target farm, such as the number of pigs and employees, to provide practical recommendations for successful implementation. This study discusses the potential benefits of an automated pig feeding system. This information will assist farmers and industry stakeholders in making informed decisions regarding the adoption of automated technologies to improve pig farming practices.

## Materials and Methods

### Ethical approval

This study does not require ethical approval as the study was not based on humans or animals.

### Study period and location

This study was conducted from January 2023 to May 2023 at Regional Smart-Center, non-profit limited company A. Baitursynov Kostanay Regional University, Kostanay, Republic of Kazakhstan.

### Study design

A simplified cost–benefit analysis was conducted to assess the economic feasibility of implementing an automated feeding system in the specific context of pig farming.

### Case study site: Pig farm in Kazakhstan

The pig farm in Kazakhstan was selected due to its geographical proximity to the research institute, which was chosen as the case study site for investigating the implementation of an automated pig feeding system. This farm is representative of the pig farming industry in Kazakhstan and provides valuable insights into the potential benefits and challenges associated with adopting automated feeding technologies in this context. The pig farm is located in the Kostanay region of Kazakhstan. The farm maintained a population of approximately 500 pigs (approximately 230 sows). At the time of the study, farm employed conventional feeding methods in which wet feed is manually distributed to pigs at regular intervals throughout the day. The farm had a workforce of 12. There was growing interest in exploring the potential of an automated feeding system to enhance feeding precision, optimize feed efficiency, and reduce labor requirements. The parameters necessary for calculating the cost–benefit analysis are presented in Table-1.

**Table-1:** Farm metrics and characteristics—selected parameters.

Parameter	Value
Feed cost per 1 kg	1 USD
Average annual feed intake per 1 pig	230 kg
Average lifespan of a pig	720 days
Average kilograms of meat obtained from a finished pig	160 kg
Average price per 1 kg of meat	3 USD
Average monthly salary per employee	400 USD

## Results

### Automated pig feeding system

**Figure-1:**
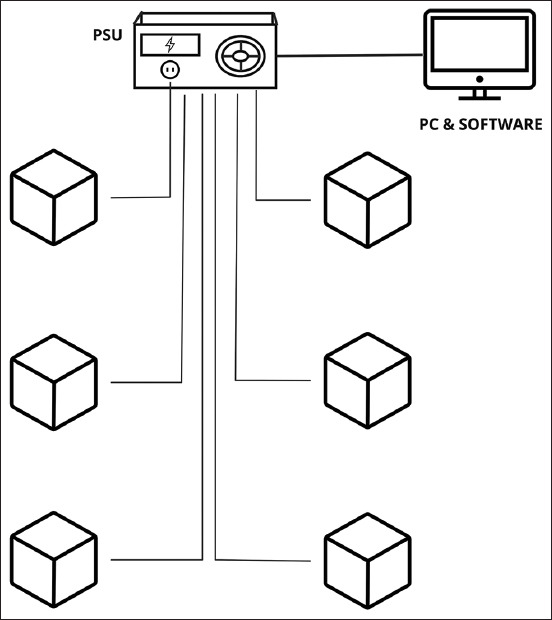
Schematic of the automated pig feeding system.

The results of this study provide an overview of an automated pig feeding system that is suitable for implementation on a selected farm in Kazakhstan based on available commercial solutions. Figure-1 shows an automated pig feeding system with several interconnected components. The core of the system comprises a central control unit that coordinates and manages the feeding process. Multiple feeding stations were connected to the control unit and strategically distributed within the pig housing area.

Each feeding station, equipped with a feed hopper or storage bin, could serve 60–80 sows by providing them with the necessary feed. The feed was delivered from the storage bin to the trough through a mechanical or pneumatic conveyance system. Only one animal had access to the trough because it was equipped with a split that could be adjusted in width. By installing scales at the entrance of the feeder, the system accurately determined the daily weight of animals using the data stored to monitor their weight dynamics.

The central control unit collects and analyzes data from weight sensors, providing valuable insights into the feeding behavior and growth of the pigs. It may also include connectivity options for remote monitoring and control, enabling the system to be managed and adjusted from a central location.

**Figure-2:**
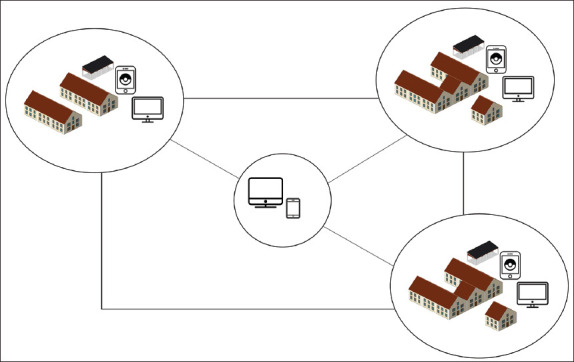
Schematic diagram of the automated control and management system of a pig farm.

Figure-2 presents an overview of the automated feeding system for pigs. Automated monitoring and control systems implemented in pig farm complexes provide real-time information and supervision for each regulator within each production hall. This system allows the monitoring of microclimate parameters, enabling printing reports that can be conveniently utilized. Furthermore, the automated feeding system for pigs operates as an integrated system that enables continuous and accurate tracking and recording of feed consumption by each animal, even within group housing. This functionality was achieved through a network of feeding stations connected to a personal computer.

Managing the system is both convenient and trouble-free because it functions through a PC interface, and remote maintenance is feasible with the aid of modern interfaces. Moreover, these production processes can be seamlessly integrated into databases to facilitate pig farming planning.

### Cost–benefit analysis

The provided cost–benefit analysis provided in Table-2 is a simplified approach that may not encompass all possible costs and benefits. This section provides an overview of the potential applications and benefits of an automated pig feeding system. The results of this basic calculation indicate that the benefits outweigh the costs, suggesting that implementing such a system may be advantageous.

**Table-2:** Simplified cost–benefit analysis.

S. No.	Cost	USD	S. No.	Benefit	USD
1	Purchase of equipment	30,000	1	Improved feed efficiency	44,000
2	Installation expenses	2,000	2	Enhanced reproductive performance	45,000
3	Maintenance and repair	1,500	3	Labor savings	17,000
4	Software	1,000			
5	Training	2,000			
	Total	36,500			106,000

## Discussion

### Cost–benefit analysis

Cost–benefit analysis plays a crucial role in evaluating the implementation and operation of an automated pig feeding system, even for pig farms with approximately 500 pigs. Although the existing literature may not provide a comprehensive cost–benefit analysis specific to such systems, valuable insights can still be derived from related research that highlights quantifiable benefits. Table-3 [11–13] presents a compilation of the potential benefits supported by reputable sources, which served as a starting point for assessing the cost–benefit analysis of the automated pig feeding system.

**Table-3:** Potential benefits of automated pig feeding systems.

Variable	Reference	Coefficient
Improved feed efficiency	Garrido-Izard *et al*. [11]	0.434 (the average feed efficiency estimation)
Enhanced reproductive performance	Automatic Feeding System of Sows-Intelligent Feeding [12]	2.4 (the average annual number of births)
Labor savings	Papandroulakis *et al*. [13]	30%–40% (decreased labor requirement for feeding)

It is worth noting that the approach adopted in this study differs from that proposed by Pham *et al*. [14], who focused on predicted calculations of pig body weight within specific timeframes using computer simulations of an automated pig feeding system. Instead, our approach aimed to provide a comprehensive perspective on the potential implementation of the system by considering the various benefits and costs associated with its adoption.

Automated pig feeding systems can address several problems and provide solutions to improve pig farming operations. The following are some common problems that an automated pig feeding system can help solve.

#### Accurate feed management and feed monitoring

Garrido-Izard *et al*. [11] conducted a study using data obtained from electronic feeding stations and reported an average feed efficiency of 0.434 for pigs. Applying this finding to farms that implement automatic feeding systems, pigs can be expected to achieve the same weight or growth with a lower amount of feed if the farm’s previous feed efficiency was lower than 0.434.

Automated feeding systems enable the precise control of the quantity and timing of feed provided to each pig. This ensures optimal nutritional levels, as highlighted by Gaillard *et al*. [3]. The capability of the system to track and monitor individual feed intake allows for improved assessment of performance and behavior, as emphasized by Arcidiacono *et al*. [9], facilitating the detection of changes in feeding behavior.

#### Enhanced animal welfare

When considering real-life companies and their experiences with automated pig feeding systems, it has been stated that the average annual number of births on a sow farm can be increased to 2.40 per year as a result of the beneficial effects of implementing the entire system [12]. Factors such as precise feed delivery, individualized feeding, optimized nutrition, and enhanced monitoring of sow health and behavior can positively affect sow fertility and productivity.

Automated feeding systems promote animal welfare by ensuring consistent access to feed, reducing stress during feeding, and minimizing the competition among pigs for food. According to Georgsson and Svendsen [15], feeding systems that create high levels of competition can have negative effects. Furthermore, Benjamin and Yik [2] suggested that remote livestock monitoring, such as through automated systems, enables the quantitative and early detection of welfare issues, allowing stockpersons to intervene promptly and address concerns.

#### Improved labor efficiency

Papandroulakis *et al*. [13] conducted a study on automatic hatchery feeding systems and estimated that such systems could reduce labor requirements by approximately 30%–40%. Although their research specifically focused on hatcheries, the principle of labor automation in feeding activities can be applied to similar automatic feeding systems for pigs. Therefore, their findings may be adopted as a reference point for understanding the potential labor reduction in an automated pig feeding system. Instead of manually feeding each pig individually, the automatic feeding system takes care of the feeding process, allowing the farm staff to focus on other important tasks.

#### Data collection and data analysis

Automated feeding systems generate a wealth of data related to feed consumption, growth rates, and feeding behaviors [2]. This wealth of information can be leveraged through effective data management and analysis, allowing farmers to make informed decisions and optimize their feeding strategies. However, it is noteworthy that the accuracy and reliability of the collected data heavily rely on the chosen technology, particularly the sensors employed [16].

## Conclusion

This study presents a design for an automated pig feeding system capable of transforming current practices owing to its ability to enhance the feeding process, optimize feed utilization, and boost overall operational efficiency. A thorough cost–benefit analysis indicates that the advantages of this innovation outweigh its costs. This suggests the feasibility of adopting such a system and emphasizes its economic prudence for farms. These findings align with and support the expanding trend toward automated pig feeding systems, signaling a considerable evolution in contemporary farming practices.

## Authors’ Contributions

GKD: Study conception and design and critical revision of the manuscript. VAM: Acquisition of data, analysis, and interpretation. ZAU and MKB: Drafted the manuscript and conducted the cost–benefit analysis. LZS: Reviewed literature, synthesized and analyzed the key findings, interpreted acquired data, and drafted the manuscript. All authors have read, reviewed, and approved the final version of the manuscript.
